# Evaluation of Antimicrobial Activity of *Aloe vera* Gel Extract on Hospital‐Acquired Methicillin‐Resistant *Staphylococcus aureus*

**DOI:** 10.1155/bmri/8787650

**Published:** 2025-09-21

**Authors:** Jones Gyamerah, Justice Kumi, Eric N. Y. Nyarko, Janet Ampofo, Sherif Hamidu, Eunice E. Ampem-Danso, Vida Yirenkyiwaa Adjei, Felicia Owusu

**Affiliations:** ^1^ Department of Pharmaceutics, Entrance University of Health Sciences, Accra, Ghana; ^2^ Department of Clinical Pathology, Noguchi Memorial Institute for Medical Research, College of Health Sciences, University of Ghana, Accra, Ghana, ug.edu.gh; ^3^ Department of Chemical Pathology, University of Ghana Medical School, College of Health Sciences, University of Ghana, Accra, Ghana, ug.edu.gh; ^4^ Department of Bacteriology, Noguchi Memorial Institute for Medical Research, College of Health Sciences, University of Ghana, Accra, Ghana, ug.edu.gh

## Abstract

**Introduction:** *Aloe vera* has a long history as a medicinal plant with numerous therapeutic uses. Methicillin‐resistant *Staphylococcus aureus* (MRSA) is a type of bacteria resistant to commonly used antibiotics such as penicillin. Hospital‐acquired methicillin‐resistant *Staphylococcus aureus* (Ha‐MRSA) refers to MRSA infections that arise in healthcare settings, usually 48 h or longer after hospital admission or in individuals with recent hospitalization. This research is aimed at investigating the effectiveness of *Aloe vera* gel extract against Ha‐MRSA.

**Methods:** The agar well boring diffusion and the broth dilution test methods were employed, respectively, to assess the susceptibility of Ha‐MRSA to *Aloe vera* gel extract in ethanol and DMSO medium. These extraction media were compared for their effectiveness as the solvent of extraction for the susceptibility test.

**Results:** A minimum inhibitory concentration of 0.2 mg/L of *Aloe vera* gel extract in the ethanolic extraction medium was achieved, indicating susceptibility of the Ha‐MRSA strain as recommended by EUCAST/ESCMID.

**Conclusion:** *Aloe vera* gel ethanol extract was sensitive against Ha‐MRSA strains but was resistant in the DMSO extraction method. It is recommended that further research be carried out to ascertain the bioactive compounds responsible for antimicrobial activity.

## 1. Introduction

*Aloe vera* (*Aloe barbadensis*) has been of scientific interest for centuries due to its diverse cosmetic and medicinal benefits of the leaves and other parts [1]. Research indicates that,*Aloe vera* and its derivatives positively impact human health when applied topically or consumed as juice or extract [2]. Several scientific studies have highlighted its wide range of biological activities, including antiviral, antimicrobial, antitumor and antifungal properties [1, 3]. The use of *Aloe vera* in traditional medicine in India, parts of South America, and Africa to treat various wounds and skin diseases suggests the potential efficacy of its gel extract phytochemical constituents as a potential antidote to methicillin‐resistant *Staphylococcus aureus* (MRSA) [4]. The overuse of antibiotics elevates the risk of antimicrobial resistance in microorganisms, presenting a significant global public health issue. Moreover, various antibiotics have proved ineffective due to bacteria resistance [5]. Several studies have investigated the potential of *Aloe vera* gel extract to inhibit *Staphylococcus aureus* growth [6–8]. MRSA is one of the most challenging antibiotic‐resistant bacteria in the world. It can cause severe infections such as bones, joints, central nervous system and serious respiratory infections [9–11]. A research conducted on molecular characterization of MRSA isolates from a hospital in Ghana reported 47% prevalence of hospital‐acquired methicillin‐resistant *Staphylococcus aureus* (Ha‐MRSA) among tested isolates and highlighted multiple resistance to multiple antibiotics [12]. Ha‐MRSA refers to MRSA infections that occur in healthcare settings, typically 48 h or more after hospital admission or in patients with recent hospitalization, surgery, or medical procedures [13]. Ha‐MRSA is a significant cause of nosocomial infections, often leading to bloodstream infections, pneumonia, and surgical site infections [13]. It is associated with higher morbidity and mortality rates due to its resistance to multiple antibiotics, making treatment challenging [10, 14]. The economic impact of antibiotic administration and resistance on individuals, healthcare systems, and governments cannot be overstated. According to [15], patients infected with antimicrobial‐resistant pathogens have greater expenses than patients infected with antimicrobial‐susceptible pathogens [15]. Antimicrobial resistance is a serious public health concern in Ghana, because it is complicated to treat infections caused by resistant bacterial strains, which could quickly spread across the population to endemic and epidemic levels [16]. Assessing the efficacy of plant‐based medicines in traditional medicine applications is critical because these treatments have few adverse effects, are inexpensive, and are widely available [17]. The growth and spread of multidrug‐resistant pathogenic bacteria have motivated research into new sources of antimicrobial drugs, such as plant metabolites [18]. A variety of plants have undergone pharmacological testing, resulting in the development of novel antibiotics [19]. Plants have advantageous multipurpose effects derived from their distinct bioactive ingredients [19]. *Aloe vera* is a promising plant with a wide range of medicinal benefits. Although *Aloe vera* has long been known for its antimicrobial properties, information on its susceptibility to Ha‐MRSA, which has become more resistant to many antibiotics, is not readily available. The findings of this research could provide preliminary data that could guide and explore the discovery of biologically active compounds that may prove effective in the treatment of Ha‐MRSA infections [11]. The present study is aimed at determining the antimicrobial activity of *Aloe vera* gel extract on Ha‐MRSA bacteria strain.

## 2. Materials and Methods

### 2.1. Collection and Preparation of *Aloe vera* Samples

The *Aloe vera* plants were gathered and procured from a specialized *Aloe vera* farm located in Kasoa (GPS‐latitude 5°33 ^′^ 27 ^″^, longitude 0°24 ^′^51 ^″^) in the Central region of Ghana. Subsequently, the plants were presented to the Pharmacognosy Department of the Entrance University of Health Sciences for authentication.

### 2.2. Sample Preparation

The fresh leaves of fully grown and healthy *Aloe vera* plants were thoroughly washed in tap water for 5 min. Subsequently, the leaves were cleaned using sterile distilled water. Then, a sterile knife was employed to dissect the leaves longitudinally, ensuring the removal of the colorless *Aloe gel* (parenchymatous tissue) while excluding the fibers. The extracted gel was then allowed to dry air in an oven set at 60 °C for 72 h. Once the gel was completely dry, it was ground into a fine powder using a mortar and pestle.

### 2.3. Dimethyl Sulfoxide Extraction

*Aloe vera* (20 g) powder was soaked in 200 mL of DMSO for 4 days at room temperature to facilitate extraction of the active components present in the *Aloe vera* gel. Dimethyl sulfoxide is utilized in *Aloe vera* extraction owing to its capacity to dissolve a diverse array of chemicals, including both polar and nonpolar molecules, hence facilitating the extraction of various bioactive constituents from *Aloe vera*. Additionally, DMSO is regarded as relatively safe for biological systems and compatible with numerous assay techniques, rendering it a flexible solvent for research applications. Following the extraction, the solution was suction filtered using Whatman filter paper No. 1, and the resulting filtrate was evaporated to dryness using a rotary evaporator, from a speed of 120 RPM and increasing up to 150 RPM within 2 h. The filtrate further underwent freeze‐drying with a lyophilizer to ensure that all moisture was removed from it. The supernatant, or the remaining liquid, was then collected and stored in a refrigerator at a temperature of 4 °C [20]. DMSO and sterile distilled water were used as negative controls. Before being utilized for antimicrobial susceptibility testing, the batch of extracts was dissolved in sterile distilled water. This step ensures the suitability of the extracts for the intended testing purposes [20].

### 2.4. Ethanolic Extraction

*Aloe vera* powder (20 g) was soaked in 200 mL of ethanol for 4 days at room temperature. Ethanol is extensively utilized in plant extraction due to its capacity to dissolve a diverse array of chemicals, encompassing both polar and nonpolar components. Following the extraction, the solution was suction‐filtered using Whatman filter paper No. 1, and the resulting filtrate was evaporated to dryness using a rotary evaporator. The filtrate further underwent freeze‐drying to ensure that all moisture was removed from it. The supernatant, or the remaining liquid, was then collected and stored in a refrigerator at a temperature of 4°C. Before being utilized for antimicrobial susceptibility testing, the batch of extracts was dissolved in sterile water. This step ensures the suitability of the extracts for the intended testing purposes [21].

### 2.5. Preparation of Agar Plate

Mueller–Hinton agar (Oxoid, CM0337) was prepared according to the manufacturer’s instructions. Briefly, 19 g of dehydrated Mueller–Hinton agar powder was weighed and dissolved in 500 mL of distilled water in a clean media bottle. The mixture was thoroughly mixed by swirling and then heated on a hot plate until the agar was completely dissolved. The medium was sterilized by autoclaving at 121°C for 15 min. After autoclaving, the agar was cooled to approximately 60°C, then aseptically poured into sterile Petri dishes (20 mL per plate) inside a laminar flow clean hood. The plates were allowed to solidify at room temperature, inverted and checked for sterility after 24 h of incubation at 37°C before use [22].

### 2.6. Preparation of Inoculum

A fresh pure culture of Ha‐MSRA was obtained by subculturing a single colony onto nutrient agar, followed by incubation at 37°C for 24 h. Subsequently, about 3–5 single colonies of Ha‐MSRA were transferred into a test tube containing 5‐mL sterile Mueller–Hinton broth medium (Oxoid, CM0337B) prepared based on the manufacturer’s instructions. Thorough mixing was conducted to disperse the bacteria. The inoculated broth culture was then vortexed to mix and incubated at a temperature of 37°C for 24 h to facilitate bacterial growth. The turbidity of the optical density was then assessed and adjusted to 0.5 McFarland standard (approximately 1–2 × 10^8^ CFU/mL) using a Nephelometer (BD PheonixSpec, 440910, United States) to ensure a consistent inoculum density [23].

## 3. Susceptibility Test

### 3.1. Inoculation of Agar Plate

*Staphylococcus aureus* subsp. *aureus* ATCC 25923 strain was used as a control strain for susceptibility testing to *Aloe vera* gel extract [24]. A sterile swab was immersed in the inoculum container, making sure that the swab was not excessively wet. The dry surface of the Mueller–Hinton agar plate was then inoculated by streaking the swab three times across the entire agar surface. The plate was then rotated around 60 degrees after each streak to ensure uniform distribution of the inoculum. Following the disposal of the swab into a proper container, the inoculated agar plate was allowed to air dry at room temperature for 5 min before proceeding to the next stage [24].

### 3.2. Boring of Wells for the Plant Extract

A cork borer with a diameter of 6 mm was utilized to create wells on the agar plate that had been inoculated. For each of the Ha‐MRSA strains, a total of seven wells were bored on the surface of the agar. Among these wells, five contain varying concentrations of the plant extract of 20%, 40%, 60%, 80%, and 100%, respectively. One well contained a concentration of the positive control (oxacillin), and the remaining well contained the negative control (sterile distilled water). It was ensured that the wells were not bored closer than 24 mm from each other, measured from center to center. Following that, the petri dishes were appropriately marked and labeled. Subsequently, 4 mL of each sample was pipetted into their respective wells [24]. The plates were placed in an incubator at a temperature of 37°C for 24 h. Following that, the plates were removed from the incubator, and the zones of inhibition were measured [23].

### 3.3. Incubation of Agar Plates

#### 3.3.1. Measurement of Zone of Inhibition

Following the incubation period, the zone of inhibition was assessed visually utilizing a ruler to determine the measurement to the closest millimeter (mm). The measurements were conducted on the lower surface of the plates, specifically gauging the diameter of the zones surrounding the plant extracts and the positive control, respectively [23].

#### 3.3.2. Broth Culture

Following the inoculation of the colonies with a loop, the growth was moved to Mueller–Hinton broth which was prepared with manufactures instructions. The broth was incubated at a temperature of 37°C until it reached a turbidity corresponding to 0.5 McFarland. The prepared inoculum was subsequently diluted in broth to achieve a final organism concentration of 5 × 10^5^ CFU/mL. The dilution process ensured that the desired density of microorganisms was attained for further experimentation. From the stock extract solution (100%; 100 mg/L), 1 mL was combined with 1 mL of the 0.5 McFarland standard, resulting in a concentration of 0.1 mg/L for the first series of dilutions and measurements. Subsequently, Dilutions were also made for 20%, 40%, 60%, 70%, 80%, and 100%, respectively [25].

## 4. Results

### 4.1. Susceptibility Test of *Aloe vera* Gel Extract on Ha‐MRSA

Tables 1 and 2 shows the broth dilution test of *Aloe vera* gel extract in DMSO, while Tables 3 and 4 shows results from the ethanol medium extraction. The minimum inhibitory concentration (MIC) of the *Aloe vera* gel extract was expressed in milligrams per liter as a standard reporting system stated by [26]. The *Aloe vera* gel extract in DMSO was resistant to Ha‐MRSA in the broth dilution testing method (Tables 1 and 2). Ethanolic *Aloe vera* gel extract inhibited Ha‐MRSA with an MIC from 0.02, 0.04, 0.05, 0.06, and 0.07 mg/L (Table 3) until complete inhibition occurred at 0.2, from 0.08, 0.09, 0.1, and 0.12 mg/mL (Table 4). Tables 5 and 6 show results of zones of inhibition of *Aloe vera* gel extract in DMSO and ethanol in the agar diffusion method. *Aloe vera* gel extract in both the DMSO and ethanol medium were resistant against Ha‐MRSA bacteria strain at a concentration up to 100%. However, the antibiotic standard drug use was resistant to Ha‐MRSA in both the broth and the agar medium.

**Table 1:** MICS of *Aloe vera* gel extract in DMSO (20%–70%) against Ha‐MRSA with the broth dilution method.

**Bacterial strains/antimicrobial agent MICS**	**20% 320** mg**/L**	**40% 300** mg**/L**	**50% 280** mg**/L**	**60% 250** mg**/L**	**70% 240** mg**/L**	**Standard antibiotic: 4** mg**/L**
Ha‐MRSA	Resistance	Resistance	Resistance	Resistance	Resistance	Oxacillin‐resistant

*Note: Aloe vera* gel extract in DMSO was resistant to Ha‐MRSA in the broth dilution testing method.

**Table 2:** MICS of *Aloe vera* gel extract in DMSO (80%–200%) against Ha‐MRSA with the broth dilution method.

**Bacterial strains/antimicrobial agent MICS**	**80%-220** mg**/L**	**90%-200** mg**/L**	**100%-190** mg**/L**	**120%-170** mg**/L**	**200%-140** mg**/L**	**Standard antibiotic 4** mg**/L**
Ha‐MRSA	Resistance	Resistance	Resistance	Resistance	Resistance	Oxacillin‐resistant

*Note: Aloe vera* gel extract in DMSO was resistant to Ha‐MRSA in the broth dilution testing method.

**Table 3:** Intermediate MICS of *Aloe vera* gel extract in ethanol (20%–70%) against Ha‐MRSA with the broth dilution method.

**Bacterial strains/antimicrobial agent MICS**	**20%-0.02** mg**/L**	**40%-0.04** mg**/L**	**50%-0.05** mg**/L**	**60%-0.06** mg**/L**	**70%-0.07** mg**/L**	**Standard antibiotic: 4** mg**/L**
Ha‐MRSA	Sensitivity (partial)	Sensitivity (partial)	Sensitivity (partial)	Sensitivity (partial)	Sensitivity (partial)	Oxacillin‐resistant

*Note:* The ethanolic *Aloe vera* gel extract inhibited Ha‐MRSA in the broth dilution testing method.

**Table 4:** FINAL MICS of *Aloe vera* gel extract in ethanol (80%–200%) against Ha‐MRSA with the broth dilution method.

**Bacterial strains/antimicrobial agent MICS**	**80%-0.08** mg**/L**	**90%-0.09** mg**/L**	**100%-0.1** mg**/L**	**120%-0.12** mg**/L**	**200%-0.2 mg/L**	**Standard antibiotic: 4 mg/L**
Ha‐MRSA	Sensitivity (partial)	Sensitivity (partial)	Sensitivity (partial)	Sensitivity (partial)	Sensitivity (full)	Oxacillin‐resistant

*Note:* The ethanolic *Aloe vera* gel extract inhibited Ha‐MRSA in the broth dilution testing method.

**Table 5:** Zones of inhibition by *Aloe vera* gel extract in DMSO against Ha‐MRSA with the agar well diffusion method.

**Bacterial strains/zones of inhibition**	**20%-6** mm	**40%-6 mm**	**60%-6 mm**	**80%-6 mm**	**100%-6 mm**	**Standard antibiotic: 6 mm**
Ha‐MRSA	Resistance	Resistance	Resistance	Resistance	Resistance	Oxacillin‐resistant

*Note: Aloe vera* gel extract in DMSO medium was resistant against Ha‐MRSA.

**Table 6:** Zones of inhibition by *Aloe vera* extract in ethanol against Ha‐MRSA with the agar well diffusion method.

**Bacterial strains/zones of inhibition**	**20%-6 mm**	**40%-6 mm**	**60%-6 mm**	**80%-6 mm**	**100%-6 mm**	**Standard antibiotic: 6 mm**
Ha‐MRSA	Resistance	Resistance	Resistance	Resistance	Resistance	Oxacillin‐resistant

*Note: Aloe vera* gel extract in ethanol medium was resistant against Ha‐MRSA.

### 4.2. Evaluation of the Antimicrobial Activity of the *Aloe vera* Gel Extract

Various MICS were determined against the *Aloe vera* gel extract concentrations. It also shows that microbial sensitivity to the extract varies significantly among bacterial strains in the intermediate MICs and the final MIC. These differences are unlikely due to random chance, as the *F*‐test and *p* value of 0.01 against a standard *p* value of 0.05 indicate statistical significance. This shows that the *Aloe vera* gel extract was effective against the bacterial strains.

### 4.3. Statistical Analysis

The data obtained in this study were analyzed using one‐way ANOVA. Significant differences were determined at 95% (*p* < 0.05), with a *p* < 0.05 being statistically significant. A *p* value of 0.016, which was obtained, indicates a significant difference between the intermediate and final MICS *Aloe vera* gel extract’s effect on Ha‐MRSA strains as measured by MIC is statistically significant.

## 5. Discussion

Ha‐MRSA is a kind of MRSA infection that develops in healthcare facilities such as hospitals and nursing homes. It is frequently connected with invasive surgeries, extended hospital stays, and compromised immune systems [27]. Ha‐MRSA infections can lead to severe consequences, including bloodstream infections, pneumonia, and sepsis [28]. The current study is aimed at investigating the effectiveness of *Aloe vera* gel extract against the Ha‐MRSA (more virulent strain). Ha‐MRSA strains were susceptible to the *Aloe vera* gel ethanolic extract at an MIC of 0.2 mg/L (complete inhibition) (Table 4), starting from 0.02 mg/L (Table 3), using the broth dilution method. The *Aloe vera* gel extract suppressed Ha‐MRSA at a lower concentration; this renders it more effective than the findings of [29]. In a study by [29] “Synergistic Potential of Antimicrobial Combinations against MRSA”, Ha‐MRSA (clinical strain) showed resistance to oxacillin at MICs of 4 and 128 mg/L, respectively. Again, research work conducted by [28] on “Synergistic Bactericidal Activity of a Novel *β*‐Lactam Combination against MRSA,” MICS of amoxicillin ranged from 32 mg/L˃1024 mg/L and 1 mg/L˃256 mg/L for cefdinir. In the current study, the standard antibiotic, oxacillin, failed at 4 mg/L to show activity against the Ha‐MRSA strains. Ha‐MRSA strains were, however, resistant to the *Aloe vera* gel extract when the agar well diffusion method was used for susceptibility testing. According to [30], this could be due to the agar well diffusion assay method that could be affected by the differential diffusion of extract components in the aqueous media.

Ha‐MRSA strains were resistant to the DMSO *Aloe vera* gel extract (Tables 1 and 2). This can be compared to the research done by [31]. They found that DMSO extracts of three selected plants (*Calendula officinalis, Hypericum perforatum*, and *Glycyrrhiza glabra*) had lower MICS than the standard ampicillin, contrary to what was anticipated. They attributed that occurrence to the physiology of the type of plant involved, the part of the plant being used, and the quantity of extract. They also found interactions between plant phytochemicals and DMSO to be responsible [31]. According to [32], this could be due to the interaction between the *Aloe vera* gel phytochemical constituents and the DMSO. *Aloe vera* gel extract contains a wide range of bioactive compounds, including polysaccharides, enzymes, and other substances. These components can interact with other molecules in DMSO, in an antagonistic manner [20], potentially affecting their activity [31]. This led to an antagonistic effect between the molecules; hence, neither the *Aloe vera* gel phytochemical constituents nor the DMSO were released to inhibit the Ha‐MRSA strains [32].

## 6. Conclusion

From the study, Ha‐MRSA strains were susceptible to the ethanolic *Aloe vera* gel extract at the intermediate and final MIC, inhibiting the growth of the Ha‐MRSA strains using the broth dilution method. However, Ha‐MRSA strains were resistant to *Aloe vera* gel extract in DMSO medium. Further research is required to establish the bioactive agents responsible for the activity.

**Nomenclature**
MIC: minimum inhibitory concentration
Ha‐MRSA: hospital‐acquired methicillin‐resistant *Staphylococcus aureus*
Partial sensitivity: means Ha‐MRSA strain is neither fully resistant nor fully susceptible
Intermediate MIC: refers to an MIC that falls between susceptibility and resistance

## Disclosure

All the authors have read and approved the final manuscript for publication.

## Conflicts of Interest

The authors declare no conflicts of interest.

## Author Contributions

J.G. and J.K. conceptualized the study, and were involved in methodology, sample collection, laboratory analysis, supervision, data analysis, writing the original draft, reviewing and editing of the final draft. E.N.Y.N. and J. A were involved in methodology, data curation and writing, review and editing of the final draft. E.E.A‐D. and S.H. contributed to the review and editing of the final draft. F.O. was involved in methodology, laboratory analysis, and editing of the final draft.

## Funding

No funding was received for this manuscript.

## Data Availability

The data used and/or analyzed during the current study are available with this submission as in the main manuscript. All other available data will be provided upon request.

## References

[1] Surjushe A. , Vasani R. , and Saple D. , *Aloe vera*: A Short Review, Indian Journal of Dermatology. (2008) 53, no. 4, 10.4103/0019-5154.44785, 2-s2.0-58149472002. PMC2763764 19882025

[2] Sánchez M. , González-Burgos E. , Iglesias I. , and Gómez-Serranillos M. P. , Pharmacological Update Properties of Aloe vera and Its Major Active Constituents, Molecules. (2020) 25, no. 6, 10.3390/molecules25061324, 32183224. PMC7144722 32183224

[3] Catalano A. , Ceramella J. , Iacopetta D. , Marra M. , Conforti F. , Lupi F. R. , Gabriele D. , Borges F. , and Sinicropi M. S. , *Aloe* vera―An Extensive Review Focused on Recent Studies, Foods. (2024) 13, no. 13, 10.3390/foods13132155. PMC11241682 38998660

[4] Feily A. and Namazi M. R. , Aloe vera in Dermatology: A Brief Review, Giornale Italiano Di Dermatologia E Venereologia: Organo Ufficiale, Societa Italiana Di Dermatologia E Sifilografia. (2009) 144, no. 1, 85–91. 19218914

[5] Chin K. W. , Michelle Tiong H. L. , Vijitra L. I. , and Ma N. L. , An Overview of Antibiotic and Antibiotic Resistance, Environmental Advances. (2023) 11, 10.1016/j.envadv.2022.100331, 100331.

[6] Arbab S. , Ullah H. , Weiwei W. , Wei X. , Ahmad S. U. , Wu L. , and Zhang J. , Comparative Study of Antimicrobial Action of Aloe vera and Antibiotics Against Different Bacterial Isolates From Skin Infection, Veterinary Medicine and Science. (2021) 7, no. 5, 2061–2067, 10.1002/vms3.488, 33949142. 33949142 PMC8464272

[7] Chelu M. , Musuc A. M. , Popa M. , and Calderon Moreno J. , Aloe vera-Based Hydrogels for Wound Healing: Properties and Therapeutic Effects, Gels. (2023) 9, no. 7, 10.3390/gels9070539, 37504418. PMC10379830 37504418

[8] Khan R. U. , Naz S. , De Marzo D. , Dimuccio M. M. , Bozzo G. , Tufarelli V. , Losacco C. , and Ragni M. , Aloe vera: A Sustainable Green Alternative to Exclude Antibiotics in Modern Poultry Production, Antibiotics. (2023) 12, no. 1, 10.3390/antibiotics12010044. PMC9854562 36671245

[9] Pantosti A. and Venditti M. , What Is MRSA?, European Respiratory Journal. (2009) 34, no. 5, 1190–1196, 10.1183/09031936.00007709, 2-s2.0-71049118642. 19880619

[10] Jacobs A. , Hospital-Acquired Methicillin-Resistant *Staphylococcus aureus*: Status and Trends, Radiologic Technology. (2014) 85, no. 6. 25002642

[11] Gurung R. R. , Maharjan P. , and Chhetri G. G. , Antibiotic Resistance Pattern of *Staphylococcus aureus* With Reference to MRSA Isolates From Pediatric Patients, Future Science OA. (2020) 6, no. 4, 10.2144/fsoa-2019-0122, 32257376. PMC7117559 32257376

[12] Asante J. , Govinden U. , Owusu-Ofori A. , Bester L. A. , and Essack S. Y. , Molecular Characterization of Methicillin-Resistant *Staphylococcus aureus* Isolates From a Hospital in Ghana, African Journal of Clinical and Experimental Microbiology. (2019) 20, no. 3, 164–174, 10.4314/ajcem.v20i3.1.

[13] Risk Factors And Clinical Outcomes Of Hospital-Acquired MRSA Infection | IDR, Retrieved 14 June 2025, from https://www.dovepress.com/risk-factors-and-clinical-outcomes-of-hospital-acquired-mrsa-infection-peer-reviewed-fulltext-article-IDR. 10.2147/IDR.S223536 PMC6885554 31819553

[14] Walsh T. R. , Gales A. C. , Laxminarayan R. , and Dodd P. C. , Antimicrobial Resistance: Addressing a Global Threat to Humanity, PLoS Medicine. (2023) 20, no. 7, e1004264, 10.1371/journal.pmed.1004264. 37399216 PMC10317217

[15] Kim T. , Oh P. I. , and Simor A. E. , The Economic Impact of Methicillin-Resistant *Staphylococcus aureus* in Canadian Hospitals, Infection Control & Hospital Epidemiology. (2001) 22, no. 2, 99–104, 10.1086/501871, 2-s2.0-0035137682, 11232886. 11232886

[16] Yevutsey S. K. , Buabeng K. O. , Aikins M. , Anto B. P. , Biritwum R. B. , Frimodt-Møller N. , and Gyansa-Lutterodt M. , Situational Analysis of Antibiotic Use and Resistance in Ghana: Policy and Regulation, BMC Public Health. (2017) 17, no. 1, 10.1186/s12889-017-4910-7, 2-s2.0-85035789157. PMC5701378 29169340

[17] Drugs H. , Safety, Cost-Effectiveness, Regulation, Current Trends, and Future Directions | SpringerLink, Retrieved 14 June 2025 10.1007/978-3-031-28780-0_62.

[18] Nostro A. , Germano M. P. , D’Angelo V. , Marino A. , and Cannatelli M. A. , Extraction Methods and Bioautography for Evaluation of Medicinal Plant Antimicrobial Activity, Letters in Applied Microbiology. (2000) 30, no. 5, 379–384, 10.1046/j.1472-765x.2000.00731.x, 2-s2.0-0034022847, 10792667. 10792667

[19] Mothana R. A. and Lindequist U. , Antimicrobial Activity of Some Medicinal Plants of the Island Soqotra, Journal of Ethnopharmacology. (2005) 96, 177–181, References—Scientific Research Publishing. (N.D.). Retrieved 14 June 2025, from https://www.scirp.org/reference/referencespapers?referenceid=2937789. 15588668 10.1016/j.jep.2004.09.006

[20] The Art of Dissolving Plant Extracts in DMSO: Temperature as a Key Factor, Natural Plant Extract Manufacturer | Natural Color Supplier. Retrieved 14 June 2025, from https://www.plantextractwholesale.com/blog2/the-art-of-dissolving-plant-extracts-in-dmso-temperature-as-a-key-factor.html.

[21] Begum H. , Shimmi S. C. , Rowshan M. M. , and Khanom S. , Effect of Ethanolic Extract of Aloe Vera Gel on Certain Common Clinical Pathogens, Borneo Journal of Medical Sciences (BJMS). (2016) 10, no. 2, 19–25, 10.51200/bjms.v10i2.626.

[22] Aryal S. , Mueller Hinton Agar (MHA)- Composition, Principle, Preparation, Results, Uses, 2022, https://microbenotes.com/mueller-hinton-agar-mha/.

[23] Benkova M. , Soukup O. , and Marek J. , Antimicrobial Susceptibility Testing: Currently Used Methods and Devices and the Near Future in Clinical Practice, Journal of Applied Microbiology. (2020) 129, no. 4, 806–822, 10.1111/jam.14704, 32418295. 32418295

[24] Treangen T. J. , Maybank R. A. , Enke S. , Friss M. B. , Diviak L. F. , Karaolis D. K. , Koren S. , Ondov B. , Phillippy A. M. , Bergman N. H. , and Rosovitz M. J. , Complete Genome Sequence of the Quality Control Strain *Staphylococcus aureus* subsp. *aureus* ATCC 25923, Genome Announcements. (2014) 2, no. 6, e01110, 10.1128/genomeA.01110-14, 2-s2.0-85006077843. 25377701 PMC4223452

[25] Swenson J. M. and Thornsberry C. , Preparing Inoculum for Susceptibility Testing of Anaerobes, Journal of Clinical Microbiology. (1984) 19, no. 3, 321–325, 10.1128/jcm.19.3.321-325.1984, 6715509. 6715509 PMC271057

[26] Determination of Minimum Inhibitory Concentrations (MICs) of Antibacterial Agents by Broth Dilution, Clinical Microbiology and Infection. (2003) 9, no. 8, ix–xv, 10.1046/j.1469-0691.2003.00790.x. 11168187

[27] Methods for the Determination of Susceptibility of Bacteria to Antimicrobial Agents. Terminology, Clinical Microbiology and Infection. (1998) 4, no. 5, 291–296, 10.1111/j.1469-0691.1998.tb00061.x. 11864348

[28] Shuping L. L. , Kuonza L. , Musekiwa A. , Iyaloo S. , and Perovic O. , Hospital-Associated Methicillin-Resistant *Staphylococcus aureus*: A Cross-Sectional Analysis of Risk Factors in South African Tertiary Public Hospitals, PLoS One. (2017) 12, no. 11, e0188216, 10.1371/journal.pone.0188216, 2-s2.0-85034246621, 29145465. 29145465 PMC5690649

[29] Yu Y. , Huang H.-L. , Ye X.-Q. , Cai D.-T. , Fang J.-T. , Sun J. , Liao X.-P. , and Liu Y.-H. , Synergistic Potential of Antimicrobial Combinations Against Methicillin-Resistant *Staphylococcus aureus* , Frontiers in Microbiology. (2020) 11, 10.3389/fmicb.2020.01919. PMC7461988 33013731

[30] Hossain T. J. , Methods for Screening and Evaluation of Antimicrobial Activity: A Review of Protocols, Advantages, and Limitations, European Journal of Microbiology and Immunology. (2024) 14, no. 2, 97–115, 10.1556/1886.2024.00035. 38648108 PMC11097785

[31] Saeed K. , Ahmad N. , Dryden M. , Cortes N. , Marsh P. , Sitjar A. , Wyllie S. , Bourne S. , Hemming J. , Jeppesen C. , and Green S. , Oxacillin-Susceptible Methicillin-Resistant *Staphylococcus aureus* (OS-MRSA), a Hidden Resistant Mechanism Among Clinically Significant Isolates in the Wessex Region/UK, Infection. (2014) 42, no. 5, 843–847, 10.1007/s15010-014-0641-1, 2-s2.0-84918774340, 24919530. 24919530

[32] Saral A. , Antibacterial Activity of DMSO Extracts of Selected Plants Against Antibiotic Resistant Clinical Isolates, Erzincan Üniversitesi Fen Bilimleri Enstitüsü Dergisi. (2019) 12, no. 2, 576–584, 10.18185/erzifbed.451871.

